# Supplementary material for: Moonless night sky increases Isistius species (cookiecutter shark) and live human contact
Source: PLoS One. 2024 Feb 21;19(2):e0291852. doi: 10.1371/journal.pone.0291852 (PMC10880985; doi:10.1371/journal.pone.0291852)
Supplement: S1 File — (PDF) [file pone.0291852.s002.pdf]

May 8, 2023

The Queen's Medical Center

Office of Research and Development

Policy on Clinical Case Reports and Case Series

I. Scope of Policy:

This policy provides guidance about the regulatory requirements associated with the presentation or publication of case reports.

II. Definition:

A clinical case reports is an educational activity involving a presentation or publication that documents an unusual or particularly interesting clinical phenomenon of a patient. It details the patient's history, signs, symptoms, test results, diagnosis, prognosis, treatment and clinical course of interesting clinical cases. It typically is performed by retrospective review of the medical record, contains a short literature review and discusses the importance of the case.

III. Case Reports and RIRC Review

1. Case reports (with three or fewer patients) generally do not meet the federal definition of human subjects research, as they are not designed to develop generalizable knowledge.[1] Therefore, **Research Institutional Review Committee** (**RIRC)** ***approval is not required.***[2-7]
2. Case series (four or more patients) do meet the federal definition of human subjects research. Therefore, **RIRC approval is required.**

IV. Case Reports and HIPAA Requirements

Although case reports do not require RIRC review, they must still comply with the HIPAA Privacy Rule requirements about Protected Health Information (PHI). Authors should follow one of the following three paths prior to working on the case study.

1) De-identify: Authors who fully de-identify the case report do not need to obtain further authorization. *Also see System Wide Policy "Uses and Disclosures of De-Identified Health Information and Limited Data Sets, SW-21-036."*
   a. Remove all 18 identifiers specified in the Privacy Rule (Appendix 1).[7]
   b. Ensure that no photo, image, video or illustration can lead to identification (including PHI embedded in the file)
   c. Ensure case described is not so unique as to be identifiable through a unique characteristic, in combination with other information, or if someone looked at public sources (e.g., media accounts)
2) Obtain HIPAA Authorization: Authors who cannot completely de-identify the case report should obtain a signed HIPAA compliant authorization form (Appendix 2 or download from intranet here) from the patient or legally authorized representative as appropriate (e.g., patient deceased).
   a. This authorization form ***does not need to be submitted to the RIRC but should be uploaded into the patients CareLink record*** (author should ask manager, director or medical director if help is needed).
3) Comply with "Case Presentations" requirements in System Wide Policy "*Use/Disclosure of Protected Health Information for Educational Purposes, SW-22-025.*"

VII. Publication Requirements

Authors who are asked by a journal or other entity to provide documentation that the case report was approved by the RIRC or did not require RIRC review may present this Policy as evidence that case reports do not require RIRC approval. Some journals may require that the institution provide written attestation that the authorization of the subject has been obtained prior to publication of the case report. To obtain this attestation, submit your case report to rirc@queens.org and a formal letter will be issued.

References

1. Basic HHS Policy for Protection of Human Subjects, 45 C.F.R. §46.101 (2018). Accessed on 5/9/23 at https://www.hhs.gov/ohrp/sites/default/files/revised-common-rule-reg-text-unofficial-2018-requirements.pdf
2. Sayre JW, Toklu HZ, Ye F, Mazza J, Yale S. Case Reports, Case Series - From Clinical Practice to Evidence-Based Medicine in Graduate Medical Education. Cureus. 2017 Aug 7;9(8):e1546. doi: 10.7759/cureus.1546. PMID: 29018643; PMCID: PMC5630458
3. Johns Hopkins Medicine. Office of Human Subjects Research, Policy 102.3 Organizational Policy on Single Case Reports and Case Series. 2023. https://www.hopkinsmedicine.org/institutional_review_board/guidelines_policies/organization_policies/102_3.html
4. Boston University Medical Center. Institutional Review Board. Case Reports and Case Series. 2023. https://www.bumc.bu.edu/irb/submission-requirements/special-submission-requirements/case-reports-and-case-series/
5. University of Alabama. IRB Guidance for Case Reports. 2014. https://www.uab.edu/research/home/images/IRB/Giudance/Other-Guidance/IRB_Guidance_for_Case_Report.pdf
6. University of Washington. Policies, Procedures and Guidance. Guidance Case Reports, IRB Review and HIPAA. Version 1.2. 2023. https://www.washington.edu/research/policies/guidance-case-reports-irb-review-hipaa/
7. University of California at Irvine. Human Subject Protections. Policy #2. Case Reports. https://research.uci.edu/human-research-protections/do-you-need-irb-review/case-reports/
8. https://www.hipaajournal.com/considered-phi-hipaa/

Todd B. Seto

Digitally signed by Todd B. Seto, MD, MPH
DN: cn=Todd B. Seto, MD, MPH, o=The Queen's Medical Center, ou, email=tseto@queens.org, c=US
Date: 2023.05.09 23:23:56 -10'00'

Todd B. Seto, MD, MPH
Director, Academic Affairs and Research

Appendix 1. Definition of De-Identified Data

Identifiers That Must Be Removed to Make Health Information De-Identified (2)

De-identified data are data that contain none of the 18 HIPAA identifiers. If all of the 18 identifiers are removed, the information is no longer (1) Individually identifiable, (2) PHI, and (3) subject to HIPAA's requirements

1) Names
2) Dates, except year
3) Telephone numbers
4) Geographic data
5) FAX numbers
6) Social Security numbers
7) Email addresses
8) Medical record numbers
9) Account numbers
10) Health plan beneficiary numbers
11) Certificate/license numbers
12) Vehicle identifiers and serial numbers including license plates
13) Web URLs
14) Device identifiers and serial numbers
15) Internet protocol addresses
16) Full face photos and comparable images
17) Biometric identifiers (i.e., retinal scan, fingerprints)
18) Any unique identifying number or code

Appendix 2. Authorization for Use and Disclosure of Protected Health Information

THE QUEEN'S HEALTH SYSTEMS

AUTHORIZATION FOR USE AND DISCLOSURE OF PROTECTED HEALTH INFORMATION

I authorize *__________________________________ *to release the protected health information of:*
(*Facility Name)

*Patient Name: ______________________________ Birthdate: ________________

Address: __________________________________ Phone #: ________________

To: *Name or Institution: ____

Address: _________________________ City, State, Zip: _____________________________

*Information to be disclosed:	* Purposes for Use and/or Disclosure:
Date(s) of Service: ____________________	☐ At the request of the individual
☐ Discharge Summary ☐ ER report	☐ Legal Purposes
☐ History & Physical ☐ Laboratory Results	☐ Insurance
☐ Consults ☐ X-Ray/Imaging Reports	☐ Physician follow-up
☐ Operative Reports ☐ Entire Record	☐ Other __________________
☐ Other: Please specify: ________________________	

_____ **(initial) I agree to the release of alcohol and/or drug abuse treatment information. (If I do not specifically agree, this information will not be disclosed):**

*** Unless otherwise revoked, this authorization will expire on the following date or event: __________________.**
If a date or event is not specified, this authorization will expire one year from my date of signature below.

This authorization is voluntary. I understand that I can refuse to sign this authorization and the facility will not condition my treatment, payment, enrollment or eligibility for benefits on the signing of this authorization except as allowed under federal privacy laws for: (i) research-related treatment; or (ii) health care provided solely for disclosure to a third party or (iii) health plan initial enrollment/eligibility determinations, underwriting or risk rating determinations.

I understand that I may revoke this authorization at any time by notifying the facility's Medical Records Department or The Queen's Health Systems' Privacy Officer, in writing, of my revocation. This is described in The Queen's Health Systems Notice of Privacy Practices. I understand that the revocation will not apply to any information that already was released in reliance on this authorization.

I understand that the health information released under this authorization may be re-disclosed by the recipient and may no longer be protected under federal privacy regulations.

I hereby release the facility from all liability and all claims of any nature whatsoever pertaining to disclosure of information, or of any professional opinions, findings, or recommendations as contained in the records released to or by the facility.

*Requestor: ______________________________ *___________________________
Signature of Patient or Authorized Representative — Print Name

*Relationship: ______________________________ *___________________________
(Relationship to Patient) **Complete only if requestor is not patient* — Date

* Items that MUST be completed for authorization to be valid

(Rev. 6/2020)
